# A Lineup for Next Anti-Obesity Medicines: Beyond Incretin-Based Pharmacotherapy

**DOI:** 10.3390/ph19071047

**Published:** 2026-07-07

**Authors:** Sangmin Lee, Hyeseon Song, Yeonwoo Kwon

**Affiliations:** 1 Department of Health Sciences, Graduate School, Dong-A University, Busan 49315, Republic of Korea; hyeseon56@naver.com; 2 Department of Medicinal Biotechnology, College of Health Science, Dong-A University, Busan 49315, Republic of Korea; yeonwoo1276@naver.com

**Keywords:** incretin-based drugs, novel drug targets, obesity

## Abstract

**Background/Objectives**: Obesity is a chronic and multifactorial disease, and the global prevalence of obesity-induced metabolic and systemic complications is expected to rise. Current peptide-based drugs that mainly target glucagon-like peptide-1 receptor activation have shown unprecedented efficacy in body weight reduction. Nevertheless, they show undesirable adverse effects including gastrointestinal effects, lean mass reduction, and rebound weight gain after ending pharmacotherapy. These underscore the medical need for additional anti-obesity drugs with novel pathways. **Methods**: Literature updating current anti-obesity pharmacotherapy and reporting potential drug targets and candidates in the last five years was searched across PubMed. The resulting literature was classified into working mechanisms and target receptors. **Results**: Recent research on incretin-based peptide drugs has focused on developing more convenient regimens by introducing longer-acting once-monthly injection or oral formulation. Novel anti-obesity targets other than incretin receptors have also been suggested. This narrative review summarizes recent research updates on peptide-based drugs and upcoming anti-obesity drug candidates tested in clinical and in vivo studies. **Conclusions**: Beyond the current peptide-based pharmacotherapy, novel anti-obesity drug candidates are waiting for further validation in clinical trials. When used alone or combined with the available drugs, these candidates may produce more effective and safer pharmacotherapy for obesity treatment.

## 1. Introduction

Obesity is defined as a chronic and relapsing disease by the World Health Organization [1]. In recent years, obesity prevalence has been expected to rise in many countries, where greater food sources are secured. Modern lifestyle modifications, characterized primarily by desk-bound or sedentary employment, have led to a marked reduction in habitual physical energy expenditure. This makes obesity a global public health issue, producing more than 1 billion people globally with obesity [2,3]. Obesity serves as a primary driver for the pathogenesis of other complications such as diabetes, cardiovascular diseases, obstructive sleep apnea, osteoarthritis, and even cancers [4]. Accordingly, there is a critical need for effective pharmacological options for obesity treatment.

Incretin refers to peptide hormones released from gut endocrine cells to stimulate insulin secretion [5]. Recently, incretin-based peptide drugs have been dominating in the drug market for obesity treatment due to their unprecedentedly enhanced efficacy in body weight control compared with traditional small molecule drugs. Semaglutide is a representative peptide drug for obesity treatment targeting glucagon-like peptide-1 (GLP-1) receptors [6]. It reduces body weight by decreasing food appetite [7]. Tirzepatide is another peptide drug that functions as a dual agonist for the GLP-1 receptor and the glucose-dependent insulinotropic polypeptide (GIP) receptor. This pharmacological trait is considered for its clinical efficacy in body weight control distinct from semaglutide [8].

Recent research has focused on developing next-generation peptide drugs for obesity treatment. The combination strategy of novel candidates with the currently available peptide drugs is currently subject to intense clinical investigation. Oral semaglutide formulation has been available for diabetes treatment [9], and in 2025, it was also approved for obesity treatment. The semaglutide pill provides another option for obesity patients that do not prefer injections. More recently, a non-peptide GLP-1 receptor agonist orforglipron was FDA-approved and is available as a once-daily oral administration [10]. Apparently, non-peptide small molecules for oral formulation can activate the GLP-1 receptor, suggesting the potential of developing non-peptide activators for other peptide receptors [11].

However, the current anti-obesity peptide drugs induce gastrointestinal adverse effects including nausea, vomiting, and diarrhea that make obesity patients uncomfortable during the drug therapy [12]. More importantly, significant lean mass reduction was observed either by semaglutide or tirzepatide treatment [13,14]. Although fat mass reduction is much greater than lean mass loss, there is a strong need for novel anti-obesity drugs with desirable adverse effect profiles.

This review summarizes the recent research updates on peptide-based pharmacotherapy, oral formulation development, drug combination strategy, and emerging novel anti-obesity drug targets and candidates. Since the current drug therapy has been limited to once-weekly subcutaneous injection, recent research has focused on developing ultralong-acting once-monthly injection or oral formulation. In addition, multiple drug targets have been tested for protecting lean mass while losing body weight. Targeting several peptide hormone receptors with one drug entity has also been tested to maximize body weight reduction. We aim to provide recent research updates on improving current peptide-based pharmacotherapy and on upcoming novel anti-obesity drug targets that may produce next generation anti-obesity drugs. This narrative review delivers a scientific introduction for scientists who plan to explore up-to-date anti-obesity drug targets and expand their research to developing anti-obesity drugs.

## 2. Methodology

For this narrative review, recent research and review articles on obesity drug development published in leading scientific journals such as *Science*, *Cell*, and *Nature* were retrieved in PubMed by using keywords such as obesity, obesity drug target, anti-obesity and so on. We focused on the articles published in the last five years from 2022 to 2026. In addition, a web-based search and Gemini free version were used to obtain recent updates on the results of clinical trials and the up-to-date information posted by pharmaceutical companies. Scientific posters and oral presentations revealed at professional meetings were also included, if available.

## 3. Research Updates on Current Peptide-Based Pharmacotherapy

### 3.1. Ultralong-Acting Peptide Drugs for Once-Monthly Injections

Peptide-based drug candidates show a short biological half-life of generally less than an hour. Protraction of the peptidal half-life in human bodies is required for clinical application. Current peptide drugs for obesity treatment are modified with lipidation (also known as fattigation) [15]. Adding a long fatty acid to peptide drugs has shown significant protraction of a peptide half-life, enabling once-weekly injections for clinical efficacy [15]. For better patient compliance, once-monthly injections with consistent efficacy have been tested in clinical trials [16,17].

An ultralong-acting GLP-1R agonist maridebart cafraglutide (MariTide) was tested in clinical trials as once-monthly (once every four weeks) subcutaneous injections [16,18]. MariTide is a peptide antibody conjugate that performs dual actions by activating GLP-1 receptors and by antagonizing GIP receptors [18]. It consists of a human monoclonal antibody that targets GIP receptors for inhibition and two GLP-1 analog peptides attached to the antibody for GLP-1 receptor activation. An in vitro functional study clearly showed that MariTide performed dual actions for the human and murine GLP-1 and GIP receptors [18]. Interestingly, GIP receptor antagonism by MariTide appears paradoxical for body weight loss since tirzepatide, a dual activator for GLP-1 and GIP receptors, is used for obesity treatment. Since GIP is essential for lipid production in adipose tissue, the concern of lipodystrophy by antagonizing GIP receptors abundant in adipose tissue was raised [19]. A further safety profile with long-term treatment of MariTide will address the potential health risk mediated by GIP receptor antagonism. The role of the GIP receptor in food appetite regulation in the brain will be discussed in a later section.

A Phase I clinical trial with MariTide showed that its half-life as an intact form (GIP receptor antibody with two GLP-1 analogs) was 14 to 16 days and its half-life as a total including a GIP receptor antibody with or without GLP-1 analogs was 21 to 24 days. These suggest that once-monthly injections are feasible for MariTide. The clinical results also supported the efficacy of once-monthly MariTide injections for body weight control. The highest dose of 420 mg of MariTide produced maximal 14.5% body weight reduction at three months after once-monthly treatment [18]. A recent Phase II clinical trial with once-monthly MariTide treatment produced 12.3% to 16.2% body weight reduction from baseline at week 52 in the obesity cohort [16]. At a 420 mg dose, vomiting and nausea were the common adverse effects shown in the treatment cohort, ranging from 14% to 24% of the cohort, although having a four-week dose escalation period reduced the frequency of these adverse effects to 2% to 6% of the cohort [16].

Another ultralong-acting GLP-1 receptor agonist, PF-08653944, was developed by Pfizer [17]. PF-08653944 (PF’3944, also known as MET-097i) is known as a GLP-1 receptor agonist for once-monthly maintenance dosing by subcutaneous injection. Molecular details on what enabled once-monthly dosing of PF’3944 have not been disclosed yet. A Phase II clinical trial showed up to 12.3% placebo-adjusted weight loss at week 28 by PF’3944. The trial consisted of two steps: once-weekly dosing with two titration steps until week 12 and once-monthly dosing from week 13 to week 28. This trial is currently ongoing through week 64, and the currently available data suggest the continued weight loss by once-monthly injections of PF’3944. Gastrointestinal adverse effects such as nausea and vomiting were also observed with PF’3944 treatment.

### 3.2. Semaglutide Pill for Oral Administration

Semaglutide for oral formulation has been approved in the United States (US) as Rybelsus^®^ for type II diabetes treatment in 2019 [20]. This is the first oral formulation of a GLP-1 receptor agonist. Semaglutide is a modified 31-amino acid peptide analog of human GLP-1(7–37). Since endogenous GLP-1 is rapidly degraded by dipeptidyl peptidase-4 (DPP-4), semaglutide has α-aminoisobutyric acid at position 8 to protect from the rapid degradation mediated by DPP-4. It also has a C18-fatty diacid at position 26 to enhance its biological half-life for once-weekly subcutaneous injection.

When semaglutide is formulated with the absorption enhancer sodium *N*-[8-(2-hydroxybenzoyl)aminocaprylate] (SNAC), the tablet with semaglutide is dissolved in the stomach, where most semaglutide is absorbed transcellularly [21]. In contrast to intestinal absorption, where the massive surface area dominates the absorption rate of drug molecules, SNAC confines semaglutide absorption to the stomach. SNAC protects semaglutide degradation by buffering low pH in the stomach and transiently enhances semaglutide absorption through the cell membrane. Interestingly, a small molecular change in SNAC markedly diminished its absorption-enhancing effect. In addition, SNAC did not help the stomach absorption of another GLP-1 receptor agonist liraglutide (a peptide-based drug similar to semaglutide). These suggest that the absorption-enhancing effect of SNAC is compound-specific and that a delicate match between a delivery molecule and an absorption enhancer is necessary. Other oral delivery options through buccal and sublingual routes might also be explored with SNAC for enhancing absorption of peptide drugs.

Oral semaglutide (Wegovy^®^ pill) has also been approved in the US for the treatment of obesity in late 2025 [22]. Nevertheless, some instructions should be strictly followed to maximize drug absorption, such as having an empty stomach in the morning before administration and refraining from any food consumption for 30 min after taking the pill [23]. These generate potential disadvantages when taking semaglutide orally, indicating the need for novel obesity drugs for better patient compliance.

### 3.3. Non-Peptide Agonists for Once-Daily Oral Administration

In addition to the oral formulation of semaglutide, a non-peptide GLP-1 receptor agonist, orforglipron (Foundayo^TM^), has been recently approved in the US for once-daily administration [24]. Orforglipron (originally OWL833 or LY3502970) was developed as a small molecule activator for the GLP-1 receptor from molecular screening followed by traditional structural activity relationship studies [25]. It is a potent GLP-1 receptor agonist with partial intrinsic activity compared with endogenous GLP-1. Orforglipron potency for cAMP production mediated by the GLP-1 receptor was much weaker than the potency of the GLP-1 peptide by 9- to 80-fold when tested with different receptor expression levels [25]. Interestingly, orforglipron did not activate β-arrestin signaling by the GLP-1 receptor, making it a G protein (or cAMP-)-biased agonist. Nevertheless, orforglipron was able to induce glucose-stimulated insulin secretion and reduce food intake in non-human primates. In vivo efficacy of orforglipron was equivalent to a GLP-1 receptor agonist exenatide that was approved for diabetes treatment. Consistently with relatively weak receptor activation potency of orforglipron, a later study by Sloop et al. suggested that orforglipron was predicted to occupy a small fraction of GLP-1 receptors (1.7%) and that this was sufficient to yield the full biological response in vivo [11]. Clinical trials with orforglipron showed significant body weight reduction by daily oral administration, and it is currently approved for obesity treatment.

Likewise, danuglipron (PF-06882961) was developed as a small molecule activator of GLP-1 receptors from screening and optimization by Pfizer [26]. It is orally available and retains nanomolar potency for cAMP production mediated by human GLP-1 receptors [26]. However, danuglipron development has ceased due to the potential liver injury observed from a patient who participated in a clinical trial [27]. Together, orally available small molecules activating GLP-1 receptors have shown clinical efficacy for body weight reduction, challenging the traditional notion that small molecules are not suitable for activating incretin receptors.

### 3.4. Targeting Two or Three Incretin Receptors for Greater Clinical Efficacy

#### 3.4.1. Targeting GLP-1 and GIP Receptors

As mentioned in the introduction, tirzepatide (Mounjaro^®^ for diabetes treatment and Zepbound^®^ for obesity treatment) is a dual agonist for GLP-1 and GIP receptors [28,29]. It was developed by engineering GLP-1 receptor activity into the GIP sequence. The GIP receptor activation potency of tirzepatide was equal to GIP potency, while tirzepatide has 13-fold weaker potency for GLP-1 receptor activation than GLP-1 [30]. Dual agonism of tirzepatide appears to induce higher efficacy in body weight reduction than the efficacy of a selective GLP-1 receptor agonist semaglutide.

The role of the GIP receptor activation in obesity treatment has been controversial. The peptide hormone GIP is secreted from enteroendocrine cells located in the duodenum and activates GIP receptors located in pancreatic β-cells. GIP receptor activation is well known for stimulating insulin secretion [31]. GIP receptors are also expressed in the brain, and hypothalamic GIP receptor activation was reported to suppress food intake in vivo [32]. GIP receptor antagonism in the brain also produces food intake suppression (exemplified with MariTide) [18,33]. Although a hypothesis to compromise this controversy has been suggested [34], how both GIP agonism and antagonism in the brain provide a similar beneficial effect on body weight control remains to be fully addressed. In addition, GIP receptors are expressed in adipose tissue, and GIP receptor activation promotes lipid storage. This effect appears to induce weight gain by storing fat to our body. However, GIP also reduces inflammation and enhances insulin sensitivity in adipose tissue [35]. It has been hypothesized that these beneficial effects may contribute to tirzepatide’s efficacy in losing body weight by improving adipose tissue health and function [36].

Tirzepatide is also a biased agonist for GLP-1 receptor-mediated cAMP production versus β-arrestin recruitment to the receptor [29]. This trait suggests weaker ability to drive GLP-1 receptor internalization, making tirzepatide action superior to endogenous GLP-1 [29].

In addition to tirzepatide, ribupatide (KAI-9531) as a GLP-1/GIP receptor dual agonist has been initially developed by Hengrui Pharmaceuticals in China [37]. A Phase III international trial with ribupatide is ongoing by Kailera Therapeutics. A Phase II clinical trial performed in China showed significant 23.6% weight loss from baseline at week 36 by 8 mg ribupatide once-weekly injections [37]. The company expects that ribupatide can be a category-leading option for obesity treatment. Viking therapeutics is also testing a GLP-1/GIP receptor dual agonist VK2735 in clinical trials for obesity treatment [38]. Once-weekly subcutaneous injection of VK2735 showed 14.7% body weight reduction from baseline at week 13 with the highest dose tested (15 mg/week). Roche also posted updates on a GLP-1/GIP receptor dual agonist CT-388 whose 24 mg once-weekly injection showed significant placebo-adjusted weight loss of 22.5% at week 48 [39].

#### 3.4.2. Targeting GLP-1 and Glucagon Receptors

The idea of targeting both GLP-1 and glucagon receptors originated from an endogenous peptide oxyntomodulin (OXM). OXM is a 37-amino acid peptide that contains a full glucagon sequence [40]. It is generated in the gut by being cleaved from a preproglucagon peptide. Since OXM also has sequence similarity to GLP-1, OXM activates both glucagon and GLP-1 receptors despite its relatively weaker potency than endogenous peptides [41]. Nevertheless, OXM was reported to suppress appetite and to reduce food intake in humans [42]. Due to its short plasma half-life (~11 min) [43], long-acting OXM analogs were developed [41]. Having a cholesterol moiety at the C-terminus of OXM enhanced metabolic stability, and the long-acting OXM analog with the cholesterol moiety was used as a GLP-1 and glucagon receptor dual agonist. The dual agonist showed superior weight loss to a selective GLP-1 receptor activator in mice [41]. In addition, the dual agonist induced glucose tolerance comparable with a selective GLP-1 receptor agonist [41]. These results clearly suggest the potential of producing greater efficacy in body weight control by using dual agonism of the GLP-1 and glucagon receptors.

Traditionally, glucagon is well known for its hyperglycemic effects. Since GLP-1 receptor activation mediates insulin secretion and decreases blood glucose, the hyperglycemic effects of glucagon can be counteracted by GLP-1 receptor activation. Glucagon signaling is also known to play a critical role in insulin secretion [44]. In addition, glucagon inhibits food intake and controls meal size [45,46]. Accordingly, the efficacy of a GLP-1 receptor agonist is expected to be reinforced by glucagon receptor activation.

In 2025, a GLP-1/glucagon receptor dual agonist mazdutide (Xinermei^®^) has been approved for obesity treatment in China [47]. Mazdutide (also known as IBI362 or LY3305677, developed by Innovent Biologics and Eli Lilly and Company) is the peptide analog of mammalian OXM with a fatty acyl side chain to extend its half-life to being suitable for once-weekly injection. It has nanomolar affinity for human glucagon and GLP-1 receptors (K_I_ 17.7 nM and 28.6 nM, respectively) [48]. Once-weekly subcutaneous injection of mazdutide in clinical trials showed efficacy in treating diabetes and in reducing body weight [49,50]. Mazdutide 6 mg once-weekly injection showed significant 14.1% body weight reduction from baseline at week 48. A bit higher dose of mazdutide (9 mg/week) than that used in previous clinical trials (4 mg to 6 mg/week) was also reported to show 12.8% body weight reduction at week 24, although the gastrointestinal side effects were reported in more than 30% of the treatment cohort [51].

Several other investigational drug candidates are being tested in clinical trials for obesity treatment such as survodutide (BI 456906, developed by Zealand/Boehringer-Ingelheim) [52,53,54], pemvidutide (ALT-801, developed by Altimmune) [55,56,57,58], and efinopegdutide (MK-6024, developed by Merck and Hanmi Pharmaceutical) [59,60]. Since glucagon receptor activation is well known for its role in liver fat reduction [61], these GLP-1/glucagon receptor dual agonists are currently tested in clinical trials for the treatment of metabolic dysfunction-associated steatohepatitis (MASH).

#### 3.4.3. Targeting GLP-1/GIP/Glucagon Receptors

Retatrutide (LY-3437943, developed by Eli Lilly and Company) is the peptide agonist targeting three types of receptors: GLP-1/GIP/glucagon receptors. It is acylated with a fatty acid for its long-acting property and once-weekly injection [62]. When the receptor activation was measured with cAMP production, retatrutide showed 9-fold higher potency for GIP receptor activation than endogenous GIP [62]. In contrast, retatrutide showed more than 2-fold weaker activation potency for GLP-1 and glucagon receptors than the respective endogenous peptide, suggesting that it has more GIP activity than GLP-1 and glucagon receptor activation [62]. In obese mice, daily treatment of retatrutide (10 nmol/kg) showed greater weight loss than the same dose of tirzepatide [62]. Glucagon receptor activation by retatrutide was shown to increase energy expenditure [62]. The increased energy expenditure contributed to greater efficacy of retatrutide in body weight reduction than tirzepatide, which lacked glucagon receptor agonism. Recently, the results of the Phase III clinical trial with retatrutide showed significant reduction in body weight and HbA_1C_ (glycated hemoglobin representing the level of blood glucose) [63,64]. Once-weekly subcutaneous injection of 12 mg of retatrutide reduced 28.7% body weight reduction from baseline at week 68 in obesity and overweight populations with osteoarthritis [63]. For diabetes patients, it showed 16.8% body weight reduction from baseline at week 40 [64]. These results suggest the strong potential of retatrutide for treating obesity and type II diabetes [64]. Consistent with other incretin-based therapy, retatrutide also showed adverse effects such as nausea, diarrhea, and vomiting in 17% to 26% of the treatment group [64]. More clinical results of retatrutide are expected for the next year.

Novo Nordisk is also developing a triple agonist UBT251 with United Biotechnology that has shown significant efficacy for HbA_1C_ and body weight reduction in the Phase II clinical trial performed in China [65,66]. The safety and tolerability profile of UBT251 was consistent with what was previously reported with other triple agonists. Novo Nordisk expects to initiate global Phase II trials with UBT251 with diabetes and obesity populations [65].

#### 3.4.4. Benefits of Targeting Multiple Receptors

Targeting multiple incretin receptors at the same time has shown greater efficacy in body weight reduction than monotherapy. Tirzepatide is a dual agonist for GLP-1/GIP receptors, and it showed greater efficacy than a selective GLP-1 receptor agonist, semaglutide. Once-weekly injection of tirzepatide (10 mg or 15 mg/week as the maximal tolerated dose) reduced body weight from baseline by 20.2% at week 72, while semaglutide (1.7 mg or 2.4 mg/week as maximal tolerated dose) showed a 13.7% reduction [67]. The two drugs produced comparable gastrointestinal adverse effects [67].

GLP-1/glucagon receptor dual agonists also showed promising results in clinical efficacy compared with semaglutide. When the clinical efficacy of the dual agonist mazdutide (6 mg/week injection) or survodutide (1.8 mg/2 weeks injection) was compared with semaglutide (1 mg/week injection), they showed greater efficacy in reducing body weight and blood glucose than semaglutide alone [68,69]. Moreover, the triple agonist retatrutide for GLP-1/GIP/glucagon receptors showed body weight reduction by up to 28% from baseline at week 68 [63]. Unfortunately, gastrointestinal adverse effects were observed in the treatment cohorts more frequently than those shown in the placebo group.

The combination strategy of adding novel drug candidates to currently approved semaglutide or tirzepatide is used to achieve greater efficacy in body weight reduction. The leading example is CagriSema, a regimen of dual injections with cagrilintide (an amylin analog) and semaglutide. Although CagriSema showed greater body weight reduction than semaglutide alone, gastrointestinal adverse effects were still reported [70]. Thus, novel drug candidates beyond the current incretin-based drugs will be necessary to provide reduced adverse effects while achieving significant body weight loss.

### 3.5. Lean Mass Protective Agents with Semaglutide

One of the concerning adverse effects of semaglutide is lean mass reduction. A recent clinical study showed that once-weekly 2.4 mg semaglutide injection decreased about 3 kg of lean mass at month 7, which constituted 5% of total body weight of the participants [14]. Although most of the body weight reduction was from fat mass, the significant lean mass reduction indicates the strong need for preserving muscle mass during semaglutide administration.

Several agents for preserving lean body mass have been tested in obesity populations [71]. Blocking type II activin receptors (ActRIIA/B) with an investigational antibody bimagrumab was reported to produce significant muscle growth in animals and humans by inhibiting muscle degradation [72,73]. Bimagrumab has been tested with or without semaglutide co-treatment in a Phase II clinical trial [74]. The intravenous injection of bimagrumab (30 mg/kg every 12 weeks) itself was able to significantly reduce total body weight by 9.7% from baseline, while the placebo group showed only 2.5% reduction at week 48. Once-weekly 2.4 mg semaglutide injection and its combination with bimagrumab reduced total body weight by 14.8% and 20.2% from baseline at week 48, respectively. As expected, semaglutide treatment reduced lean body mass by 7.9% from baseline at week 48. However, the bimagrumab co-treatment group maintained lean body mass and showed only 2.6% reduction at week 48. This significant protection of the lean body mass suggests the potential of bimagrumab co-treatment for preserving lean mass while losing body weight. The beneficial effect of bimagrumab appears promising for testing further in a Phase III clinical trial. Table 1 summarizes the current development status of the approved drugs and potential drug candidates for obesity treatment targeting GLP-1, GIP, and/or glucagon receptors. 

## 4. Recent Anti-Obesity Drug Candidates Targeting Other Receptors

### 4.1. Amylin Receptor Activators

Amylin is a 37-amino acid peptide hormone secreted from pancreatic β-cells with insulin after meals [75]. Secreted amylin activates its receptors and provides benefits for the control of blood glucose and body weight by reducing food appetite, slowing gastric emptying, and inhibiting glucagon secretion [75]. Several amylin receptor agonists were developed as once-weekly subcutaneous injections. The most advanced drug candidate is cagrilintide, developed by Novo Nordisk [76] and its combination with a GLP-1 receptor agonist semaglutide (CagriSema). CagriSema is a combination regimen of once-weekly injections with 2.4 mg of cagrilintide and 2.4 mg of semaglutide. Recent Phase III clinical trials with CagriSema showed 20.4% body weight reduction in overweight and obesity populations and 13.7% body weight reduction in overweight or obesity populations with type II diabetes [70,77]. Novo Nordisk has filed for FDA approval of CagriSema for weight management [78], and CagriSema is expected to be approved in late 2026.

Other long-acting amylin analogs have also been tested in clinical trials [79], such as eloralintide (Eli Lilly and Company) [80], petrelintide (Zealand Pharma and Roche) [81,82], AZD6234 (AstraZeneca) [83], GUB014295 (Gubra, also recently labeled as ABBV-295 by AbbVie) [84,85], and amycretin (a single molecule with dual agonism developed by Novo Nordisk) [86].

### 4.2. Melanocortin 4 Receptor (MC4R) Agonists

MC4R belongs to the class A G protein-coupled receptors (GPCRs). MC4R is expressed in the central nervous system, where it plays a critical role in energy homeostasis and satiety [87,88]. It has been reported that MC4R loss of function was implicated in early-onset severe obesity [89], whereas gain of function mutations of MC4R were linked to low body mass index [90].

Interestingly, MC4R is known to have an agonist and an antagonist, both of which are endogenously expressed. MC4R is activated by α-melanocyte-stimulating hormone (α-MSH) for anorexic effects and is antagonized by the agouti-related peptide (AgRP) for promoting appetite [91,92]. Based on MC4R regulation of food appetite, a potent α-MSH analog setmelanotide was developed and has been approved for the treatment of obesity induced by genetic variations in MC4R [93]. Setmelanotide is a cyclic peptide consisting of eight amino acids with an internal disulfide bond [94]. Setmelanotide was 13-fold more potent than α-MSH for cAMP production mediated by MC4R [94]. A recent study also showed the binding mode of setmelanotide for MC4R that initiates satiety signaling [94]. By activating MC4R, setmelanotide decreases hunger and increases satiety. Recent studies are trying to develop oral formulation [95] and to expand the use of setmelanotide to broader obesity [96,97]. The combination with a GLP-1/GIP receptor dual agonist tirzepatide with MC4R agonists has also been tested for anti-obesity efficacy [98].

### 4.3. Dual Agonism of GLP-1 and Leptin Receptors

Adipose tissue releases leptin that circulates in blood and acts on leptin receptors in the brain. Leptin receptor activation is known to suppress food intake and to reduce body weight [99]. However, exogenous leptin rarely affects body weight in leptin-sufficient conditions such as obesity. The lack of efficacy is caused by leptin resistance, which is associated with decreased leptin receptor expression, leptin signaling dysfunction, and others [100]. Nevertheless, leptin signaling was reported to enhance body weight loss when used in combination with other therapies [101]. The recent study with tirzepatide and leptin co-administration showed synergistic effects on body weight reduction in mice [102], suggesting the potential benefit of using leptin with other anti-obesity drugs.

Polex-Wolf et al. designed a GLP-1/leptin receptor dual agonist by linking a GLP-1 receptor agonist with leptin [103]. The dual agonist markedly reduced body weight in a leptin-sensitive animal model, and the reduction was greater than the leptin conjugate with inactive GLP-1. A previous report showed that there were hypothalamic neurons marked by both GLP-1 and leptin receptor expression [104]. The dual agonist efficacy appeared to be mediated by these neurons expressing both GLP-1 and leptin receptors. Although these results were based on the in vivo model, leptin receptor signaling in the brain may be useful for producing greater anti-obesity efficacy when added to GLP-1 receptor agonist monotherapy.

### 4.4. Peptide YY (PYY)

PYY is co-secreted with GLP-1 and OXM from enteroendocrine L cells located in intestines after meals. PYY activates neuropeptide Y Y2 receptor (Y2R), which are expressed in the hypothalamus [105,106], and Y2R activation reduces food intake [107]. A recent study targeted Y2R and developed potent long-acting PYY analogs with selective Y2R affinity through extensive peptide variation screening [108]. These PYY analogs (analog number 15 and number 5) showed significant efficacy in body weight reduction in genetically mutated diabetic db/db mice [108]. When PYY analog number 15 was used with semaglutide, the combination showed body weight reduction greater than the effect of the PYY analog or semaglutide alone in diet-induced obesity mice [108]. Interestingly, the body weight reduction of the combination was largely from fat loss, and the lean mass was maintained in the mice [108].

PYY analog number 15 (PYY1875, developed by Novo Nordisk) was able to progress into clinical trials for obesity treatment. Although a PYY1875 add-on to semaglutide showed clinically meaningful body weight reduction in a Phase II clinical trial, the clinical efficacy of the combination was less than what was expected [109]. In addition, gastrointestinal-related adverse effects were commonly observed by PYY1875 treatment, and the PYY1875 doses used in the trial were not well tolerated [109]. Based on the tolerability issue from the Phase II clinical trial, it appears that Novo Nordisk holds further PYY analog development for obesity treatment [110].

### 4.5. Corticotropin-Releasing Hormone (CRH) Receptor 2 Agonism

CRH (or corticotropin-releasing factor) is secreted from the hypothalamus and is well known for its role in initiating stress responses through the hypothalamic–pituitary–adrenal axis [111]. CRH activates the corticotropin-releasing hormone receptor 1 and 2 (CRHR1 or CRHR2) to mediate its physiological effects. CRHR1 is mainly expressed in the brain, whereas CRHR2 is strongly expressed in peripheral tissue such as skeletal muscles and blood vessels [112]. CRHR2 activation in skeletal muscles is known to induce muscle hypertrophy [113] and it was also reported to reduce food intake in rodents through gastric vagal nerve activation [114,115,116]. Since lean mass loss is a concerning adverse effect of the current peptide-based therapy, CRHR2 activation has become an attractive drug target that can reduce body weight without losing lean mass.

Urocortin (UCN) is a neuropeptide that activates CRHR1/2 receptors making it belong to the CRH family [117]. UCN has three types in humans: UCN1/2/3. UCN has shown beneficial effects on the treatment of heart failure, diabetes, and obesity [118]. Since UCN2 selectively binds and activates CRHR2 compared with UCN1/3 [112], several UCN2 analogs have been developed for body weight reduction [119,120,121,122]. The most advanced candidate appears to be HM17321 developed by Hanmi Pharmaceutical. Once-weekly injections of HM17321 (0.5 mg/kg) showed promising results in obese monkeys, where a significant body weight loss was observed while lean mass was increased up to 8% from baseline at week 12 [123]. A Phase I clinical trial with once-weekly HM17321 injection is ongoing in the US to test HM17321 efficacy in body weight reduction with the hope of gaining lean mass [121].

### 4.6. Cholecystokinin (CCK) 1 Receptor Agonism

CCK belongs to gastrointestinal hormones and is known for its effects on promoting digestion, such as gallbladder emptying and pancreatic enzyme secretion [124,125]. Later studies found that CCK also promotes insulin secretion [126] and reduces food appetite through vagal nerve activation [127]. CCK elicits its effects by activating CCK 1 and 2 receptors in gastrointestinal tracts and the central nervous system. The CCK 1 receptor is responsible for stimulating insulin secretion [128], and its activation in vagal afferent nerves reduces food appetite for anti-obesity effects. CCK 2 receptors are related to gastric acid secretion and other brain functions [127]. Accordingly, CCK 1 receptor activation has been targeted for anti-obesity drug development.

Novo Nordisk developed multiple CCK 1 receptor-selective peptide agonists in 2019 based on systemic investigation of a native CCK 1 receptor ligand CCK-8 [129]. CCK-8, consisting of eight amino acids, is the shortest bioactive form of CCK that is processed from the translational product of CCK gene (Prepro-CCK) [124]. One of the developed CCK 1 receptor-selective peptides is NN9056. It showed strong food intake reduction in pigs and had a plasma half-life of 113 h in minipigs, which was enabled by the fatty acid linkage to NN9056 [129,130]. However, chronic administration of NN9056 showed the potential risk of pancreatitis and tropic actions of the exocrine pancreas in monkeys [131]. Novo Nordisk decided to halt further development of NN9056 due to the translatable risk to humans.

More recent studies developed a single molecule agonist that activates both GLP-1 and CCK 1 receptors by linking a GLP-1 analog to NN9056 [132,133]. Interestingly, the dual agonists for GLP-1 and CCK 1 receptors showed superior hypoglycemic and insulinotropic effects and superior body weight loss to NN9056 or semaglutide alone in vivo [132,133]. These results suggest that GLP-1/CCK 1 receptor dual agonism may provide a potential benefit for diabetes and obesity treatment. The dual agonist was also reported to improve cognitive performance in an Alzheimer’s disease mouse model by protecting neurons [134,135].

### 4.7. Fibroblast Growth Factor (FGF) 21

FGF21 is a protein hormone consisting of 181 amino acids produced mainly from liver and adipose tissue. FGF21 increases fatty acid oxidation and reduces lipids in liver [136]. In addition, it enhances insulin sensitivity and decreases body weight [136,137]. The physiological activity of FGF21 is mediated by the activation of the FGF receptor 1c, 2c, and 3c expressed in the liver and other tissues. In addition to FGF receptors, a co-receptor called β-klotho is required for FGF21 function by serving as a high-affinity trap for FGF21 [138]. The potential of FGF21 for treating metabolic diseases such as fatty liver, diabetes, and obesity has been investigated. Long-acting FGF21 analogs were developed and have been tested for treating MASH.

Currently leading FGF21 analogs are efruxifermin and pegozafermin [136]. Efruxifermin developed by Akero Therapeutics (recently acquired by Novo Nordisk [139]) is the fusion protein of a modified human FGF21 and the fragment crystallizable (Fc) region of human IgG_1_ [140]. This fusion protein is available as once-weekly injections for clinical trials. Weekly efruxifermin injection (70 mg/week) decreased hepatic fat fraction by 14% at week 12, while the placebo group showed a 0.3% increase [141]. Efruxifermin (50 mg/week) also improved glycemic control by increasing insulin sensitivity and induced body weight loss at week 24 [142]. However, the weight loss with efruxifermin treatment at week 24 was just 2.9 kg. These results suggest that efruxifermin is more suitable for reducing liver fat in MASH populations than for body weight loss in obesity patients.

Pegozafermin is a long-acting glyco-pegylated recombinant FGF21 analog developed by 89 Bio that was recently acquired by Roche [136,143]. Pegozafermine greatly reduced plasma triglyceride and liver fat fraction in the patients with severe hypertriglyceridemia that was characterized by high plasma triglyceride levels (above 500 mg/dL) [144]. In addition, pegozafermin once-weekly injections (30 mg) or injections every 2 weeks (44 mg) showed over 26% fibrosis improvement in nonalcoholic steatohepatitis patients at week 24, compared with only 7% in the placebo group [145]. Efruxifermin and pegozafermin are currently in Phase III clinical trials for MASH treatment [136,146,147]. Another FGF21 analog BOS-580 was developed by Boston Pharmaceuticals [136,148]. A recent Phase II clinical trial with BOS-580 reported that once-monthly subcutaneous injection of BOS-580 (300 mg/month) significantly improved liver fibrosis and increased MASH resolution compared with the placebo group [149]. Dual agonism for GLP-1 and FGF21 receptors has also been tested for treating liver fibrosis and metabolic diseases [150,151,152].

### 4.8. Growth Differentiation Factor 15 (GDF15)

GDF15 is a cysteine-rich dimeric 25 kDa protein hormone originally reported as macrophage inhibitory cytokine 1 [153]. It is widely expressed across various organs and tissues, and once expressed, it activates glial cell line-derived neurotrophic factor receptor α-like (GFRAL) to mediate its biological functions [154]. GDF15 induced weight loss in diet-induced obesity mice and in non-human primates [154]. GDF15 reduces food intake by activating GFRAL in the brain [155] and enhances energy expenditure in skeletal muscles promoting body weight reduction [156].

Since GDF15 has a short half-life (about 3 h in mice and non-human primates) [157] and a low stability due to proteolytic cleavage, multiple long-acting GDF15 analogs with improved pharmacokinetic properties were developed [158,159]. Although long-acting GDF15 analogs showed the potential for obesity treatment in animal models, they failed to produce meaningful weight loss in clinical trials [159]. LY3463251 (developed by Eli Lilly and Company) and MBL949 (developed by Novartis) both were unable to show meaningful weight loss in humans. Body weight reduction by once-weekly LY3463251 injections was modest, with only a 2.74 kg reduction at week 12 compared with the placebo group [160]. Consistently, bi-weekly injection of MBL949 produced clinically small and minimal weight loss until week 16 [161].

The fusion protein of GLP-1 and GDF15 (QL1005) was developed by Beijing QL Biopharmaceutical [162]. QL1005 showed promising and superior efficacy in body weight reduction to semaglutide in mice and produced significant body weight loss dose-dependently in non-human primates. QL1005 was also reported to decrease inflammation and fibrosis in MASH in animal models [163]. Anti-obesity and other beneficial effects of QL1005 remain to be validated in humans.

### 4.9. Ghrelin Receptor Antagonism

Ghrelin is a 28-amino acid peptide secreted from the stomach [164]. It activates ghrelin receptors (also known as growth hormone secretagogue receptors) expressed in the hypothalamus acting as a ‘hunger’ hormone [165]. Ghrelin receptor activation stimulates food appetite and promotes fat storage. In Japan, a ghrelin receptor agonist anamorelin was approved and is available to treat cancer cachexia (loss of appetite) by stimulating food intake [166]. In contrast, ghrelin receptor antagonism has been targeted for obesity treatment.

Liver-expressed antimicrobial peptide 2 (LEAP2) is an endogenously expressed 40-amino acid peptide that antagonizes the ghrelin receptor [167]. LEAP2 fully inhibited ghrelin receptor activation and blocked the major effects of ghrelin in mice such as stimulating food intake. A recent clinical trial reported that LEAP2 intravenous infusion (infusion rate 40 pmol/kg/min for 320 min) to non-diabetic men with obesity significantly reduced ad libitum food intake by 12% compared with the placebo group [168]. In addition, LEAP2 infusion limited postprandial glucose elevation, suggesting its anti-diabetic efficacy. Although this early stage-clinical trial had some limitations (such as excluding females from the study), this proof-of-concept study provided evidence that LEAP2 may be useful for obesity and diabetes treatment. A long-acting LEAP2 analog consisting of 11 amino acids with chemical modifications was recently reported [169], and the combination of the long-acting LEAP2 analog and other anti-obesity molecules has been investigated for body weight reduction in vivo [169,170].

### 4.10. Adenosine Receptor Agonists

While adenosine is well known as an inhibitory neurotransmitter, its receptor activation has been involved in cancer, inflammation, cardiovascular and respiratory systems, and metabolic regulation [171]. Adenosine activates four types of adenosine receptors: A1, A2A, A2B, and A3. These receptors belong to the class A GPCR and are expressed in diverse areas in humans [171]. Adenosine receptor activation was reported to mediate glucose and lipid metabolism [172]. Human A3 adenosine receptors (A3ARs) are expressed mainly in lung and liver [173], and its activation also reduces inflammation in the diseased liver [174]. Previous studies have highlighted the therapeutic potential of A3AR agonists for MASH and obesity treatment [174,175,176].

Namodenoson (CF102) is a selective small molecule A3AR agonist that has demonstrated anti-obesity effects [175]. Namodenoson treatment reduced body weight by 6.1% in diet-induced obesity mice. The anti-obesity effects of namodenoson appear to be mediated by adiponectin upregulation that is associated with improved insulin sensitivity and anti-inflammation [175]. Since adenosine receptor activation can reduce liver fat, a clinical study tested the efficacy of namodenoson in MASH treatment [177]. This Phase II clinical study also reported body weight reduction by namodenoson. Unfortunately, namodenoson (25 mg twice a day) showed a modest 2.1 kg reduction from baseline at week 12. Can-Fite BioPharma is organizing several clinical trials with namodenoson for the treatment of MASH and some cancers [178].

### 4.11. Adrenergic Agonism Biased for GPCR Kinases (GRKs)

GRKs activated by the β2-adrenergic receptor (β2AR) are known to mediate glucose uptake in muscles [179]. Nevertheless, β2AR agonists are considered unsuitable for clinical candidates to control blood glucose due to G protein/cAMP-induced cardiac side effects and β-arrestin-dependent receptor desensitization. Generally, a receptor expressed in cells can trigger multiple signaling pathways. Activating one signaling pathway selectively over others occurs in cells [180]. The ligands that selectively trigger one pathway activation are called functionally selective or biased agonists. Thus, GRK-selective (GRK-biased) β2AR agonists were developed and were tested for diabetes and obesity treatment [181].

GRK-biased β2AR agonists were found through extensive pharmacophore screening [181]. Several biased agonists demonstrated the significant glucose control in mouse models, with reduced cardiovascular side effects compared with conventional β2AR agonists. When one of the biased agonists (Compound **15**) was combined with liraglutide (a GLP-1 receptor agonist), the biased agonist reversed muscle atrophy induced by liraglutide treatment in mice [181]. This result suggests that Compound **15** may be able to preserve lean mass while reducing body weight. Compound **15** progressed to a Phase I clinical trial where once-daily dosing with 2.5 mg for up to 28 days was well tolerated in healthy volunteers and type II diabetes patients [181]. Future Phase II clinical trials would reveal the clinical efficacy of Compound **15** in the treatment of type II diabetes and obesity.

### 4.12. Growth Differentiation Factor-8 (GDF8) and Activin A (ActA)-Blocking Antibodies

GDF8 (or myostatin) and ActA have been reported as two major ligands that mediate the muscle-minimization actions of ActRIIA/B [182,183]. As mentioned earlier, bimagrumab that blocks ActRIIA/B showed significant protection from lean mass loss mediated by semaglutide [74]. Whether direct blocking of either GDF8 or ActA can prevent muscle loss mediated by a GLP-1 receptor agonist was examined. As expected, blocking two ligands together with the respective antibody (trevogrumab for GDF8 and garetosmab for ActA) prevented muscle loss and even increased muscle mass from baseline in vivo during GLP-1 receptor agonist administration [184]. A Phase I clinical trial showed that using two types of antibodies led to greater muscle growth than using either antibody alone and that the increase in muscle mass was accompanied by fat reduction [185]. A Phase II clinical trial is ongoing, and the interim results confirmed the therapeutic potential of the antibodies for improving the quality of semaglutide-induced weight loss [186]. These results suggest that the combining blockage of GDF8 and ActA may be a promising therapeutic approach for obesity management without muscle loss.

### 4.13. Small Interfering RNA (siRNA)-Based Therapy Targeting the INHBE Gene

Extensive gene sequencing studies reported that the loss of function of the *INHBE* gene was associated with favorable fat distribution [187,188]. Inhibin βE (or hepatokine activin E), the protein product of the *INHBE* gene, is expressed in liver, and it promotes adipose fat storage [189]. Blocking inhibin βE function may contribute to the treatment of metabolic diseases related to fat distribution and storage. The clinical trial with ARO-INHBE (siRNA that blocks the expression of inhibin βE) is ongoing to test the safety and efficacy of siRNA-based therapy [190]. The clinical trial is sponsored by Arrowhead Pharmaceuticals. Table 2 summarizes the current development status of anti-obesity drugs or drug candidates targeting others than GLP-1/GIP/glucagon receptors. In addition, Table 3 compared anti-obesity clinical efficacy of the drugs and drug candidates described in the present study. 

### 4.14. Early-Stage Novel Preclinical Drug Targets and Candidates for Obesity Treatment

There are early-stage drug targets and candidates whose anti-obesity effects were reported in preclinical studies. Genome-wide association and genetic mutagenesis studies have produced several key proteins that are associated with protection against obesity. Inhibition of glutamate receptor scaffolding proteins (PSD-95 and PICK1) [198] and deficiency of G protein-coupled receptor 75 (GPR75) [199] showed weight-lowering effects and obesity protection, while the loss of function of neuronal G protein-coupled receptor 45 (GPR45) [200] and of serotonin 2C receptors [201] caused obesity.

Long-acting agonists for neurokinin 2 receptor reduced body weight by suppressing appetite centrally and by increasing energy expenditure peripherally [202]. An odor receptor Or5v1/Olfr110 was identified as the oxylipin receptor, and the receptor activation by either an oxylipin 12(S)-hydroxyeicosapentaenoic acid or a synthetic receptor agonist (HOR1-C59) reduced obesity and improved glucose homeostasis [203]. An exercise-induced myokine irisin expression in mice ameliorated obesity by inhibiting inflammation, enhancing the survival of T cells, and inducing IL-33 expression in adipose tissue [204]. The nanoemulsion loaded with the X-box binding protein 1 inhibitor KIRA6 and α-Tocopherol impeded obesity progression by inhibiting adipose tissue expansion [205]. Several compounds such as annonaceous acetogenins mimic AA005 [206], artesunate [207], mitochondrially targeted tamoxifen [208], a nitroalkene derivative of salicylate (SANA) [209], and adipose-targeted triiodothyronine [210] were also reported for anti-obesity efficacy in vivo.

## 5. Conclusions

Incretin-based pharmacotherapy has revolutionized the global obesity drug market with its unprecedented high efficacy in body weight reduction compared with traditional small molecule drugs. Nevertheless, gastrointestinal adverse effects and loss of lean mass have been the concerns of the GLP-1-based therapy. Recent research has focused on developing ultralong-acting peptide drugs suitable for once-monthly injections and on preserving and further increasing muscle mass by regulating cell signaling for muscle growth.

**Figure 1:**
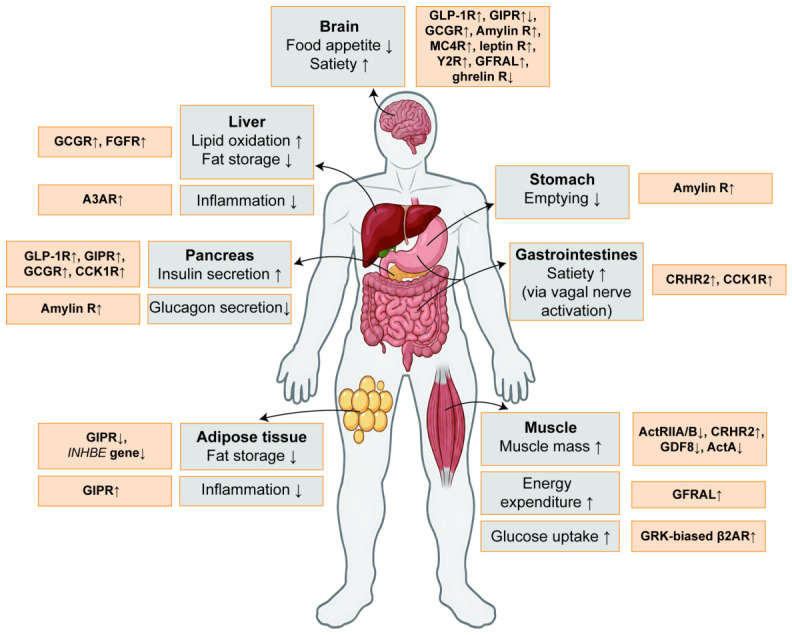
Target receptors of current anti-obesity drugs and potential drug candidates. Yellow boxes indicate the target receptors that mediate physiological changes, which are shown in gray boxes. Arrows (↑↓) in the yellow and gray boxes indicate the increase or decrease in receptor or ligand activity and in physiological changes. GDF8 and ActA are the ligands for ActRIIA/B, and direct antibodies for GDF8 and ActA were developed as anti-obesity drug candidates. Refer to the abbreviation section for abbreviations used in Figure 1.

Novel anti-obesity drug targets have been reported, and corresponding drug candidates are being tested in preclinical or clinical studies. Figure 1 summarizes potential anti-obesity drug targets and their physiological effects for body weight reduction. Decreasing food appetite and increasing satiety centrally or peripherally appear to be the main drug target. In addition, stimulating lipid oxidation and decreasing inflammation in liver and adipose tissue have been targeted, and several novel drug candidates regulating the activity of GCGR, FGFR, A3AR, and the INHBE gene are extensively tested for their clinical efficacy. Targeting GCGR and FGFR is also of interest since their activation significantly improves liver function in MASH. Current anti-obesity drugs can decrease muscle mass while mostly reducing fat mass. Protecting muscle mass is also necessary for obesity patients with diabetes since muscles can uptake glucose from blood vessels. Accordingly, drug targets that can protect and even enhance muscle mass have been reported, and drug candidates targeting CRHR2, ActA, and GDF8 are currently being tested in preclinical and clinical trials.

Losing body weight turns out to be beneficial for other diseases beyond diabetes and obesity. Peptide-based anti-obesity drugs have expanded their indications to cardiovascular diseases and obstructive sleep apnea, and their efficacy has been tested for treating chronic kidney disease, MASH, substance use disorder, and others [211]. In addition, personalized pharmacotherapy based on personal genetic information or drug response histories may be developed in the future by analyzing personal health information with the help of artificial intelligence. We hope that more effective and safer anti-obesity pharmacotherapy targeting incretin or non-incretin receptors will be available in the coming years and that in the future, the global epidemiological trend will show a marked decrease in the burden of obesity.

## Figures and Tables

**Table 1:** Current anti-obesity pharmacotherapy and drug candidates targeting GLP-1, GIP, and/or glucagon receptors.

Drugs or Candidates	Mechanisms	Developer	Status (Product, Year)	Reference
Semaglutide	Long acting GLP-1R agonist	Novo Nordisk	Approved (Wegovy^®^, 2021)	[6]
Tirzepatide	Dual GLP-1R/GIPR agonist	Eli Lilly	Approved (Zepbound^®^, 2023)	[28]
Oral semaglutide	Oral GLP-1R agonist with SNAC	Novo Nordisk	Approved (Wegovy^®^ pill, 2026)	[22]
Orforglipron	Non-peptide GLP-1R agonist	Eli Lilly	Approved (Foundayo^TM^, 2026)	[10,24]
Mazdutide	Dual GLP-1R/GCGR agonist	Innovent Biologics, Eli Lilly	Approved in China (Xinermei^®^, 2025)	[47]
Maridebart Cafraglutide (MariTide)	Ultralong-acting GLP-1R agonist + GIPR antagonist	Amgen	Phase II done and Phase III ongoing	[16]
PF-08653944 (PF’3944, MET-097i)	Ultralong-acting GLP-1R agonist	Pfizer	Phase II done	[17]
Ribupatide (KAI-9531)	Dual GLP-1R/GIPR agonist	Hengrui Pharmaceuticals, Kailera Therapeutics	Phase II done and Phase III ongoing	[37]
VK2735	Dual GLP-1R/GIPR agonist	Viking Therapeutics	Phase II done	[38]
CT-388	Dual GLP-1R/GIPR agonist	Roche	Phase II done	[39]
Survodutide (BI 456906)	Dual GLP-1R/GCGR agonist	Zealand Pharma, Boehringer-Ingelheim	Phase II and III done	[52,53,54]
Pemvidutide (ALT-801)	Dual GLP-1R/GCGR agonist	Altimmune	Phase II done for MASH and AUD	[56]
Efinopegdutide (MK-6024)	Dual GLP-1R/GCGR agonist	Merck, Hanmi Pharmaceutical	Phase II done for MASH	[59,60]
Retatrutide (LY-3437943)	Triple GLP-1R/GIPR/GCGR agonist	Eli Lilly	Phase III done	[64]
UBT251	Triple GLP-1R/GIPR/GCGR agonist	Novo Nordisk, United Biotechnology	Phase II done	[65]
Bimagrumab (+semaglutide)	Antibody targeting type II activin receptors	Eli Lilly	Phase II done	[74]

GLP-1R, glucagon-like receptor-1 receptor; GIPR, glucose-dependent insulinotropic polypeptide receptor; SNAC, sodium *N*-[8-(2-hydroxybenzoyl)aminocaprylate]; GCGR, glucagon receptor; MASH, metabolic dysfunction-associated steatohepatitis; AUD, alcohol use disorder.

**Table 2:** Potential anti-obesity drug candidates targeting others than GLP-1/GIP/glucagon receptors.

Drugs or Candidates	Mechanisms	Developer	Status	Reference
Cagrilintide	Amylin receptor agonist	Novo Nordisk	Phase III done	[70]
CagriSema	Amylin receptor agonist (Cagrilintide) + GLP-1R agonist (Semaglutide)	Novo Nordisk	Phase III done, filed for FDA approval	[70,78]
Eloralintide	Amylin receptor agonist	Eli Lilly	Phase II done	[80]
Petrelintide	Amylin receptor agonist	Zealand Pharma, Roche	Phase II done	[82]
AZD6234	Amylin receptor agonist	AstraZeneca	Phase II done	[83]
GUB014295 (ABBV-295)	Amylin receptor agonist	Gubra, AbbVie	Phase I done	[85]
Amycretin	Dual Amylin receptor/GLP-1R agonist	Novo Nordisk	Phase II done	[86]
Setmelanotide	MC4R agonist	Rhythm Pharmaceuticals	Approved for genetic obesity (Imcivree^®^, 2020)	[93]
Dual GLP-1/leptin receptor agonist	Dual GLP-1/leptin receptor agonist	Novo Nordisk	Preclinical	[103]
PYY1875	Y2R agonist	Novo Nordisk	Phase II done, development halted due to tolerability issues	[109,110]
HM17321	CRHR2 agonist	Hanmi Pharmaceutical	Phase I ongoing	[121]
NN9056	CCK 1R agonist	Novo Nordisk	Preclinical, development halted due to translatable risk to humans	[130,131]
Dual GLP-1R/CCK 1R agonist	Dual GLP-1R/CCK 1R agonist	Novo Nordisk	Preclinical	[132,133]
Efruxifermin	FGF21 analog	Akero Therapeutics, Novo Nordisk	Phase II done, Phase III ongoing for MASH	[146]
Pegozafermin	Glyco-pegylated FGF21 analog	89bio, Roche	Phase II done, Phase III ongoing for MASH	[147]
BOS-580	FGF21 analog	Boston Pharmaceuticals	Phase II done for MASH	[149]
LY3463251	GDF15 analog	Eli Lilly and Company	Phase I done (limited efficacy)	[160]
MBL949	GDF15 analog	Novartis	Phase II done (limited efficacy)	[161]
QL1005	GLP-1/GDF15 fusion protein	Beijing QL Biopharmaceutical	Preclinical	[162]
LEAP2 infusion	Endogenous ghrelin receptor antagonist	Englund et al.	Phase I done	[168]
LA-LEAP2	Lipidated LEAP2 analog	Holm et al.	Preclinical	[169]
Namodenoson (CF102)	A3AR agonist	Can-Fite BioPharma	Phase IIb ongoing for MASH	[178]
Compound **15**	GRK-biased β2AR agonist	Motso et al.	Phase I done	[181]
Trevogrumab	Anti-GDF8 antibody	Regeneron Pharmaceuticals	Phase I done, Phase II ongoing	[185,186]
Garetosmab	Anti-activin A antibody	Regeneron Pharmaceuticals	Phase I done, Phase II ongoing	[185,186]
ARO-INHBE	Inhibin βE-suppressing siRNA	Arrowhead Pharmaceuticals	Phase I/IIa ongoing	[190]

GLP-1R, glucagon-like peptide-1 receptor; MC4R, melanocortin 4 receptor; Y2R, neuropeptide Y Y2 receptor; CRHR2, corticotropin-releasing hormone receptor 2; CCK 1R, cholecystokinin 1 receptor; FGF21, fibroblast growth factor 21; GDF15, growth differentiation factor 15; LEAP2, liver-expressed antimicrobial peptide 2; LA-LEAP, long-acting LEAP; A3AR, adenosine A3 receptor; GRK, GPCR (G protein-coupled receptor) kinases; β2AR, β2-adrenergic receptor; GDF8, growth differentiation factor-8; siRNA, small interfering RNA.

**Table 3:** Recent clinical efficacy of current drugs and candidates in body weight reduction and their adverse effect profiles.

Drugs or Candidates	Patient Populations	Dose ^1^	Treatment Weeks	Efficacy (%) ^2^	Adverse Effects	References
Semaglutide (Wegovy^®^)	Overweight or obesity ^3^	2.4 mg, QW, SC (w/16 weeks DE)	68	14.9	GI effects	[191]
Tirzepatide (Zepbound^®^)	Overweight or obesity ^3^	15 mg, QW, SC (w/20 weeks DE)	72	20.9	GI effects	[192]
Oral semaglutide (Wegovy^®^ pill)	Overweight or obesity ^3^	25 mg, QD, Oral (w/12 weeks DE)	64	13.6	GI effects	[193]
Orforglipron (Foundayo^TM^)	Overweight or obesity ^3^	36 mg, QD, Oral (w/20 weeks DE)	72	11.2	GI effects	[194]
Mazdutide (Xinermei^®^)	Overweight or obesity ^4^	6 mg, QW, SC (w/8 weeks DE)	48	14.01	GI effects	[49]
Maridebart Cafraglutide (MariTide)	Overweight or obesity ^3^	420 mg, Q4W, SC (w/12 weeks DE)	52	16.2	GI effects	[16]
	Obesity with diabetes ^5^	420 mg, Q4W, SC		12.3		
PF-08653944 (PF’3944, MET-097i)	Overweight or obesity ^3^	4.8 mg, Q4W, SC (w/12 weeks DE)	28	12.3 ^6^	GI effects	[17]
Ribupatide (KAI-9531)	Overweight or obesity ^7^	8 mg, QW, SC	36	23.6 ^8^	N.A.	[37]
VK2735	Overweight or obesity ^3^	15 mg, QW, SC	13	14.7 ^9^	GI effects	[38]
CT-388	Overweight or obesity ^3^	24 mg, QW, SC (titration to 24 mg)	48	18.3	GI effects	[39]
Survodutide (BI 456906)	Overweight or obesity ^10^	4.8 mg, QW, SC (w/20 weeks DE)	46	14.9 ^9^	GI effects	[53]
Pemvidutide (ALT-801)	Overweight or obesity ^3^	2.4 mg, QW, SC	48	15.6 ^11^	GI effects	[58,195]
Efinopegdutide (MK-6024)	NAFLD ^12^	10 mg, QW, SC (w/8 weeks DE)	24	8.5 ^13^	GI effects	[59]
Retatrutide (LY-3437943)	Obesity or overweight with OA	12 mg, QW, SC (w/16 weeks DE)	68	20.0	GI effects	[63]
	Type II diabetes		40	15.3	GI effects	[64]
UBT251	Overweight or obesity ^4^	2~6 mg, QW, SC	24	19.7 ^8^	Similar to incretin-based drugs	[66]
	Type II diabetes			9.8 ^8^		[65]
Bimagrumab alone	Overweight or obesity ^3^	30 mg/kg, Q12W, IV alone	48	8.6	Muscle spasms, diarrhea, nausea, acne	[74]
Bimagrumab + semaglutide		Bimagrumab + semaglutide 2.4 mg, QW, SC		16.4		
Cagrilintide	Overweight or obesity ^3^	4,5 mg, QW, SC (w/6 weeks DE)	26	10.6	GI effects	[196]
CagriSema	Overweight or obesity ^3^	Cagrilintide 2.4 mg, QW, SC + semaglutide 2.4 mg, QW, SC (w/16 weeks DE)	68	20.4	GI effects	[70]
	Overweight or obesity with Type II Diabetes			13.7		[77]
Eloralintide	Overweight or obesity ^3^	9 mg, QW, SC	48	17.5	GI effects	[80]
Petrelintide	Overweight or obesity ^7^	QW, SC ^14^ (w/16 weeks DE)	42	10.7 ^8^	GI effects	[82]
GUB014295 (ABBV-295)	Adults with BMI < 30	2~14 mg, QW, SC	12	9.79 ^15^	GI effects	[85]
Amycretin	Overweight or obesity ^16^	0.3 to 60 mg, QW, SC (DE with 4-week intervals to 36 weeks)	36	24.3 ^17^	GI effects	[86]
Setmelanotide	Severe obesity by genetic mutations	0.5~3.0 mg, QD, SC (w/2-week interval DE to 3.0 mg)	48	12.5~25.6 ^18^	Injection site reaction and hyperpigmentation	[197]
PYY1875 + semaglutide	Obesity ^19^	Semaglutide 2.4 mg QW, SC + PYY1875 1.0 mg QW, SC ^20^	48	5.25 ^21^	GI effects	[109]
Efruxifermin	Adults with NASH ^22^	50 mg, QW, SC	24	No apparent effects	Diarrhea and nausea	[142]
Pegozafermin	Adults with NASH ^22^	15 or 30 mg, QW, SC or 40 mg, Q2W, SC	24	No apparent effects	Diarrhea and nausea	[145]
BOS-580	Adults with BMI at least 27 and MASH ^22^	300 mg, Q4W, SC	24	No apparent effects	GI effects	[149]
LY3463251	Healthy participants with elevated BMI (27 to 40)	9 mg, QW, SC (w/4 weeks DE)	12	No apparent effects	Nausea and emesis	[160]
MBL949	Obese participants with or without Type II diabetes ^23^	7.5 mg, Q2W, SC (w/6 weeks DE)	16	No apparent effects	GI effects	[161]
Namodenoson (CF102)	Adults with NAFLD ^24^	25 mg, BD, Oral	12	No apparent effects	Musculoskeletal disorders and infections	[177]
Garetosmab	Healthy postmenopausal females and males ^25^	10 mg/kg, Q4W, IV	5~16	No apparent effects	Muscle spasms, aphthous ulcer, and headache	[185]
Garetosmab + Trevogrumab	Healthy postmenopausal females ^25^	Garetosmab 10 mg/kg, Q2W, IV + Trevogrumab 6 mg/kg, Q2W, IV	6	No apparent effects	Muscle spasms and infection	[185]

^1^ A maximal dose tested in the clinical trial is shown. ^2^ Percent body weight reduction from baseline achieved by the indicated dose during the treatment period. Clinical efficacy analyzed with treatment regimen/policy estimand (traditional intention-to-treat analysis, with effects assessed regardless of treatment discontinuation or rescue intervention) is shown. ^3^ Body mass index (BMI) at least 30 or BMI 27 to 30 with at least one obesity-related complication excluding diabetes. ^4^ Adults with obesity or who were overweight with at least one weight-related coexisting condition. Obesity and overweight were defined according to Chinese criteria (BMI of ≥28 and of 24 to <28, respectively). ^5^ BMI of 27 or more and an established diagnosis of type 2 diabetes. ^6^ Placebo-adjusted weight loss analyzed as efficacy estimand, hypothetically assuming that the treatment was administered as intended for all participants (hypothetical strategy estimand). ^7^ Details on participants are not available. ^8^ Analyzed with efficacy estimand. ^9^ Percent change in body weight from baseline analyzed by a mixed model for repeated measures (MMRM) in the modified intent-to-treat population. ^10^ BMI of at least 27 without diabetes. ^11^ Least squares (LS) mean weight loss from baseline (percentage) analyzed by MMRM assuming subjects remained on treatment for the entire duration. ^12^ With liver fat content more than 10%. ^13^ LS mean change from baseline based on the longitudinal data analysis model. ^14^ Dose information is not available on the reference article. ^15^ LS mean from baseline (percentage) estimates were derived using MMRM; the participants were required to adhere to the dosing plan, and those unable to continue treatment were withdrawn from the study with no further efficacy data collected. ^16^ Adults with a BMI of 27.0–39.9. Participants with a glycated hemoglobin (HbA1c) of 6.5% or greater were excluded. ^17^ Percentage weight change was analyzed with MMRM with treatment as a factor and baseline body weight (kg) as a covariate. ^18^ Mean percent body weight changes from baseline. A small sample size was planned due to being rare diseases. Refer to the reference article for detail. ^19^ BMI 30.0–45.0 with HbA1c < 6.5%. ^20^ Semaglutide 2.4 mg (QW, SC) including 8-week DE was given for 32 weeks. After 32 weeks, PYY1875 1.0 mg (QW, SC) including 8~10-week DE with 2-week intervals was added on to semaglutide for 16 weeks. ^21^ Analyzed by using an analysis of covariance (ANCOVA) model with randomized treatment as factors and baseline body weight (kg) as a covariate. ^22^ Including F2 or F3 (indicating moderate or severe fibrosis) and an NAFLD activity score (NAS) of 4 or higher scores of at least 1 in each of steatosis, ballooning, and lobular inflammation, confirmed by biopsies. ^23^ BMI over 32. ^24^ Defined as hepatic steatosis ≥ 10%, as determined by magnetic resonance imagining-determined proton-density fat-fraction, and serum ALT ≥ 60 IU/L. ^25^ BMI 18–32. QW, once a week; SC, subcutaneous injection; GI, gastrointestinal; DE, dose escalation; QD, once-daily; Q4W, once every 4 weeks; N.A., not available; NAFLD, nonalcoholic fatty liver disease; OA, osteoarthritis; Q12W, once every 12 weeks; NASH, nonalcoholic steatohepatitis; Q2W, once every 2 weeks; MASH, metabolic dysfunction-associated steatohepatitis; BD, twice a day; IV, intravenous injection.

## Data Availability

No new data were created or analyzed in this study. Data sharing is not applicable to this article.

## Abbreviations

The following abbreviations are used in this manuscript:

GLP-1	Glucagon-like peptide-1
GIP	Glucose-dependent insulinotropic polypeptide
MariTide	Maridebart cafraglutide
US	United States
DPP-4	Dipeptidyl peptidase-4
SNAC	Sodium *N*-[8-(2-hydroxybenzoyl)aminocaprylate]
OXM	Oxyntomodulin
MASH	Metabolic dysfunction-associated steatohepatitis
ActRIIA/B	Type II activin receptors
MC4R	Melanocortin 4 receptor
GPCRs	G protein-coupled receptors
α-MSH	α-melanocyte-stimulating hormone
AgRP	Agouti-related peptide
PYY	Peptide YY
Y2R	Neuropeptide Y Y2 receptor
CRH	Corticotropin-releasing hormone
UCN	Urocortin
CCK	Cholecystokinin
FGF	Fibroblast growth factor
GDF15	Growth differentiation factor 15
GFRAL	Glial cell line-derived neurotrophic factor receptor α-like
LEAP2	Liver-expressed antimicrobial peptide 2
A3AR	A3 adenosine receptors
GRKs	GPCR kinases
β2AR	β2-adrenergic receptor
GDF8	Growth differentiation factor-8
ActA	Activin A
siRNA	Small interfering RNA
GPR75	G protein-coupled receptor 75
GPR45	G protein-coupled receptor 45
SANA	A nitroalkene derivative of salicylate
